# Co-ultramicronized Palmitoylethanolamide/Luteolin in the Treatment of Cerebral Ischemia: from Rodent to Man

**DOI:** 10.1007/s12975-015-0440-8

**Published:** 2015-12-26

**Authors:** Carlo Caltagirone, Carlo Cisari, Carlo Schievano, Rosanna Di Paola, Marika Cordaro, Giuseppe Bruschetta, Emanuela Esposito, Salvatore Cuzzocrea, Stroke Study Group

**Affiliations:** Fondazione Santa Lucia IRCCS, Via Ardeatina, 306-00179 Rome, Italy; Dipartimento di Scienze della Salute, Amedeo Avogadro University of Eastern Piedmont, Novara, Piedmont Italy; Department of Biological and Environmental Sciences, University of Messina, Viale Ferdinando Stagno d’Alcontres, no. 31, Messina, 98166 Italy; University of Padua, Padua, Italy

**Keywords:** Palmitoylethanolamide, Luteolin, Co-ultramicronization, Neuroinflammation, Neuroprotection, Brain ischemia reperfusion injury

## Abstract

Acute ischemic stroke, the most frequent cause of permanent disability in adults worldwide, results from transient or permanent reduction in regional cerebral blood flow and involves oxidative stress and inflammation. Despite the success of experimental animal models of stroke in identifying anti-inflammatory/neuroprotective compounds, translation of these putative neuroprotectants to human clinical trials has failed to produce a positive outcome. Tissue injury and stress activate endogenous mechanisms which function to restore homeostatic balance and prevent further damage by upregulating the synthesis of lipid signaling molecules, including *N*-palmitoylethanolamine (PEA or palmitoylethanolamide). PEA exerts neuroprotection and reduces inflammatory secondary events associated with brain ischemia reperfusion injury (middle cerebral artery occlusion (MCAo)). Here, we examined the neuroprotective potential of a co-ultramicronized composite containing PEA and the antioxidant flavonoid luteolin (10:1 by mass), nominated co-ultraPEALut. The study consisted of two arms. In the first, rats subjected to MCAo and treated with co-ultraPEALut post-ischemia showed reduced edema and brain infract volume, improved neurobehavioral functions, and reduced expression of pro-inflammatory markers and astrocyte markers. In the second arm, a cohort of 250 stroke patients undergoing neurorehabilitation on either an inpatient or outpatient basis were treated for 60 days with a pharmaceutical preparation of co-ultraPEALut (Glialia®). At baseline and after 30 days of treatment, all patients underwent a battery of evaluations to assess neurological status, impairment of cognitive abilities, the degree of spasticity, pain, and independence in daily living activities. All indices showed statistically significant gains at study end. Despite its observational nature, this represents the first description of co-ultraPEALut administration to human stroke patients and clinical improvement not otherwise expected from spontaneous recovery. Further, controlled trials are warranted to confirm the utility of co-ultraPEALut to improve clinical outcome in human stroke.

## Introduction

Stroke is the second leading cause of death and the preeminent cause of neurological disability worldwide [1–3]. Ischemic stroke results from the sudden decrease or loss of blood circulation to an area of the brain, resulting in a corresponding loss of neurological function. Deficits can include partial paralysis, difficulties with memory, thinking, language, and movement. While the prompt restoration of blood flow to the ischemic tissue is the current strategy of choice in clinical stroke treatment, this can cause additional damage and exacerbate neurocognitive deficits. Inflammation is a key feature of cerebral ischemia [4, 5] with major immune system players, namely microglia [6, 7] and mast cells [8, 9] acting as early responders. This response leads to the production of pro-inflammatory mediators and infiltration of other inflammatory cell populations (e.g., neutrophils, T cell subsets, monocyte/macrophages) into the ischemic brain tissue. Additional, late-phase responders include reactive astrocytes and the resulting glial scar which forms in the boundary zone of the ischemic core and may play a critical role, both in detrimental and beneficial terms [10].

Experimental animal models of stroke enabled the identification of a wide array of anti-inflammatory/neuroprotective compounds, including anti-epileptics, inhibitors of inducible nitric oxide synthase and kinases, minocycline, antioxidants, and polyphenols [11–15]. Some of these putative neuroprotectants have been tested in human clinical trials, but they have yielded little positive outcome [3, 16]. Nevertheless, neuroprotection studies utilizing animal models still provide useful insights into strategies for limiting stroke severity as they continue to offer translational potential for improving future stroke outcome [3, 17]. The failure of therapies targeted only to neuronal cell protection may be attributable, in part, to a lack of concomitant protection of cerebral blood vessels from secondary injury by inflammation and reactive oxygen species/reactive nitrogen species. Although anti-inflammatory approaches have proven successful in animal models of stroke [18, 19], attempts to translate this into clinical application have fallen short of expectations [20, 21].

In response to tissue injury and stress, the body is known to respond by producing molecules “on demand” which function to restore homeostatic balance and prevent further damage [22]. Among these is a class of lipid signaling molecules, the *N*-acylethanolamines (NAEs) [23, 24]. One NAE, in particular *N*-palmitoylethanolamine (PEA or palmitoylethanolamide), is surrounded by a large number of observations supporting its role in maintaining cellular homeostasis by acting as a mediator of resolution of inflammatory processes [25, 26]. These past years have witnessed a continually growing number of studies confirming the anti-neuroinflammatory and neuroprotective actions of PEA [27–30]. Interestingly, several recent studies have shown that a co-ultramicronized PEA/luteolin composite (co-ultraPEALut, 10:1 mass ratio) is more efficacious than PEA alone [31, 32], including animal models of spinal cord injury [33] and traumatic brain injury [34].

Based on the above observations, we carried out a two-part study with co-ultraPEALut in cerebral ischemia. In the first part of the study, we analyzed the neuroprotective and anti-inflammatory properties of co-ultraPEALut in a rat model of middle cerebral artery occlusion (MCAo), while in the second part, the effects of co-ultraPEALut (Glialia®) were assessed in stroke patients undergoing rehabilitation therapy. We chose to conduct the study in subacute-phase stroke patients, given that even at this stage, certain characteristic pathological features, in particular neuro-inflammatory processes, are still active and able to cause continued neuronal cell damage. Moreover, many patients with ischemic stroke, despite optimal medical treatment received during the acute phase, often fail to recover (or only partially), leading to persistent disability requiring rehabilitation. As Glialia® is already a marketed product, we investigated whether treatment with Glialia®, carried out simultaneously with rehabilitation therapy, can bring about a better functional recovery in stroke patients in the subacute phase over a prolonged time frame.

## Materials and Methods

### Middle Cerebral Artery Occlusion

Male Wistar rats (Harlan, Italy) weighing 270–290 g were used. Animals were housed in groups of three and kept on a 12-h light/dark cycle, under standardized temperature, humidity, and light conditions with free access to food and water. Animal care and use followed directives of the Council of the European Community (86/609/EC). All efforts were made to minimize animal suffering and to reduce the number of animals used. Focal cerebral ischemia was induced by transient MCAo in the right hemisphere. The rats were anesthetized with 5.0 % isoflurane (Baxter International) and spontaneously inhaled 1.0–2.0 % isoflurane in air by the use of a mask. Body core temperature was maintained at 37 °C with a heating pad and was monitored via an intrarectal type T thermocouple (Harvard, Kent, UK). The rats were placed in a stereotaxic system (Kopf). Middle cerebral carotid artery (MCA) occlusion was performed by inserting a 4-0 nylon monofilament (Ethilon; Johnson & Johnson, Somerville, NJ, USA), pre-coated with silicone (Xantopren; Heraeus Kulzer, Germany) and mixed with a hardener (Omnident, Germany), via the external carotid artery into the internal carotid artery to block the MCA, as originally described by Longa et al. [35] and modified by Melani et al. [36]. Sham rats were subjected to the same surgical procedure, and the filament was inserted into the internal carotid artery and immediately withdrawn. At the end of the surgical procedure, anesthesia was discontinued and the animals were returned to a prone position. Recovery from anesthesia took 15 min; animals were then allowed free access to food and water.

Regional cerebral blood flow (rCBF) was monitored by laser Doppler flowmetry (PeriFlux System 5000; Perimed AB, Stockholm, Sweden) with the use of a flexible probe over the skull as described earlier [37]. The rats that did not show a CBF reduction of at least 70 % were excluded from subsequent experiments. Cranial temperature was maintained at 36.8–37.5 °C with a heating pad. Some physiologic parameters including cranial temperature, arterial pH, PaCO_2_, PaO_2_, and glucose were measured in six additional rats. Arterial blood samples were taken before ischemia (baseline), during ischemia, and after ischemia for gases and plasma glucose measurements (Table 1).

**Table 1:** Physiological parameters, mean ± SD (*n* = 8)

Time point	Temperature (°C)	pH	PaCO_2_ (mmHg)	PaO_2_ (mmHg)	Glucose (mmol/l)
Baseline	37.2 ± 0.3	37.32 ± 0.2	38.2 ± 3.9	104.5 ± 10.9	4.31 ± 0.31
During ischemia	37.0 ± 0.2	37.36 ± 0.3	38.5 ± 5.1	103.2 ± 8.1	4.25 ± 0.22
After ischemia	36.8 ± 0.3	37.33 ± 0.1	38.3 ± 4.2	102.9 ± 9.9	4.27 ± 0.19

#### Animal Groups and Treatments

The rats were randomly allocated to the following groups:

- *Ischemia/reperfusion* + *vehicle*: the rats (*n* = 20) were subjected to MCAo (2 h) followed by 24 h reperfusion [38]. Carboxymethyl cellulose in saline (1.5 %, *w*/*v*) was administered (os) 1 h after ischemia and 6 h after reperfusion, and the rats were sacrificed 24 h later for evaluation of histological damage (*n* = 10) and Western blot (*n* = 10);
- *Ischemia/reperfusion* + *co-ultraPEALut*: the rats (*n* = 20) were subjected to surgical procedures as described above. Co-ultraPEALut (1 mg/kg in 1.5 % carboxymethyl cellulose, os) was administered (os) 1 h after ischemia and 6 h after reperfusion, and the rats were sacrificed 24 h later for evaluation of histological damage (*n* = 10) and Western blot (*n* = 10);
- *Sham* + *vehicle*: the rats (*n* = 20) were subjected to identical surgical procedures except for MCA occlusion and were kept under anesthesia for the duration of the experiment. The animals were treated (os) with 1.5 % (*w*/*v*) carboxymethyl cellulose in saline at the same time point as the MCAo group and sacrificed 24 h later for evaluation of histological damage (*n* = 10) and Western blot (*n* = 10);
- *Sham* + *co-ultraPEALut*: the rats (*n* = 20) identical to sham-operated rats except for administration of co-ultraPEALut (1 mg/kg in 15 % carboxymethyl cellulose, os) 1 h after ischemia and 6 h after reperfusion were sacrificed 24 h later for evaluation of histological damage (*n* = 10) and Western blot (*n* = 10).

In a separate set of experiments, 10 animals from each group were observed until 24 h after MCAo to evaluate motor behavior, perform neurological testing, and evaluate infarct damage (Table 2). The dose (1 mg/kg) of co-ultraPEALut, administration route (os), and vehicle used were based on our previous study [39].

**Table 2:** Effect of co-ultraPEALut (1 mg/kg, os, 1 h after ischemia and 6 h after reperfusion) on neurological score and ischemic brain damage evaluated 24 h after transient MCAo

Treatment	Neurological score	Cortical damage (mm^3^)	Striatal damage (mm^3^)
Vehicle	7.57 ± 0.21*	57.26 ± 2.33^§§^	24.28 ± 2.37^§^
Co-ultraPEALut	9.13 ± 0.23^#^	37.72 ± 1.26	12.23 ± 2.1
Sham operated	17.5 ± 0.15	0	0

Data are the mean ± SD of *n* of rats (neurological score and brain damage, *n* = 10). The volume of the ipsilateral hemisphere was as follows (mean ± SD): 127.75 ± 3.59 mm^3^ in co-ultraPEALut-treated rats. One-way ANOVA followed by Bonferroni post hoc test was performed for neurological score

*p < 0.001, versus sham-operated rats; ^#^ *p* < 0.05, versus MCAo + vehicle (cortical and striatal damage was analyzed using unpaired Student’s *t* test); ^§^ *p* < 0.05; ^§§^ *p* < 0.01, versus co-ultraPEALut-treated rats

#### Motor Behavior

About 1 h after MCAo, the rats showed spontaneous turning behavior that was evaluated every 15 min for 3–4 h post-MCAo, as described by Melani et al. [40]. Motor activity was assessed in sham-operated and vehicle- and drug-treated rats. After being placed in a round cage, the rat began rapid unidirectional walking along the perimeter of the cage and to chase its tail. The number of complete rotations was recorded manually. Five separate counting periods of 3 min each, separated by 15-min intervals, were made. Values are reported as mean rotation number/h during the five counting periods. The same rats were also evaluated after awakening and 24 h later for failure to extend fully the left forepaw and for contralateral turning when pulled by the tail. Under this condition, all the rats showed a clear circling to the left side.

#### Neurological Scoring

Neurological evaluation of motor sensory functions was carried out prior to and 24 h after MCAo by an examiner blinded to the procedure, always between 10:00 a.m. and 11:00 a.m. to exclude behavioral changes based on circadian rhythm. The neurological examination consisted of six tests [41]: (i) spontaneous activity, (ii) symmetry in limb movements, (iii) forepaw outstretching, (iv) climbing, (v) body proprioception, and (vi) response to vibrissae touch. The six test scores were summed to generate an assigned final score, which ranged from a minimum of 3 to a maximum of 18.

#### Histological Evaluation of Infarct Damage

After evaluation of motor behavior and neurological deficit 24 h post-MCAo, the vehicle-treated (*n* = 10), co-ultraPEALut-treated (*n* = 10), and sham-operated rats (*n* = 10) were randomly selected and analyzed for ischemic damage using published histological methods [36]. The rats were anesthetized and sacrificed by decapitation. The brains were rapidly removed and fixed with Carnoy’s solution (6:3:1 absolute ethanol, chloroform, and glacial acetic acid) and then embedded in paraffin after dehydration in graded series of ethanol and xylol. Coronal sections (7–8 μm) were collected at 1-mm intervals at eight different levels through the striatum (from +2.2 Bregma to −4.8 Bregma, corresponding to the ischemic area) and were stained with acetate cresyl violet (1 %) or hematoxylin and eosin (H&E). The lesioned area was viewed under light microscopy (Axiostar Plus equipped with AxioCam MRc, Zeiss) as a pale zone lacking acetate cresyl violet staining, a refection of the extent of unlabeled necrotic neurons 24 h after MCAo [41]. The specific regions included the cortical areas and hippocampus. In each region, the degree of neuronal cell injury was assessed according to an incremental 5-point scale: 0 is normal, 1 denotes 10 % selective neuronal injury, 2 denotes 10 to 50 % neuronal injury, 3 denotes 50 % neuronal injury, and 4 indicates confluent areas of pan-necrosis as previously described [42].

Tissue damage was not found in either sham-operated rats or the hemisphere contralateral to the ischemic side. Ischemic tissue damage was clearly seen in the vascular territory supplied by the MCA, i.e., the sensorimotor cortex and striatum. The pallid area was measured by utilizing an image analysis system (Image-Pro Plus; Image & Computer, Milan, Italy). Striatal and cortical damage was calculated in cubic millimeters. All histological studies were performed in a blinded fashion.

#### Quantification of Infarct Volume

The rats were anesthetized with ketamine and decapitated, and the brains were carefully removed and cut into five coronal slices of 2 mm in thickness. Slices were incubated in a 2 % solution of 2,3,5-triphenyltetrazolium chloride (TTC) at 37 °C for 30 min and immersion fixed in 10 % buffered formalin solution. TTC stains viable brain tissue red, while infracted tissue remains unstained [43]. For quantification of infracted area and volumes, the brain slices were photographed using a digital camera (Canon 4×; Canon Inc., China) and image analysis was performed on a personal computer with ImageJ for Windows [44]. To compensate for the effect of brain edema, the corrected infarct area equals the left hemisphere area minus (right hemisphere area minus infarct area) [45]. The corrected total infarct volume was calculated by summing the infarct area in each slice and multiplying by slice thickness (2 mm).

#### Immunohistochemical Localization of Chymase, Tryptase, and Glial Fibrillary Acidic Protein

Twenty-four hours after ischemia, tissues were fixed in 10 % (*w*/*v*) phosphate-buffered formaldehyde and 7-μm sections were prepared from paraffin-embedded tissues. After deparaffinization, endogenous peroxidase was quenched with 0.3 % (*v*/*v*) hydrogen peroxide in 60 % (*v*/*v*) methanol for 30 min. The sections were permeabilized with 0.1 % (*w*/*v*) Triton X-100 in phosphate-buffered saline (PBS) for 20 min. Nonspecific adsorption was minimized by incubation in 2 % (*v*/*v*) normal goat serum in PBS for 20 min. Endogenous biotin or avidin binding sites were blocked by sequential incubation for 15 min with biotin and avidin, respectively. Sections were then incubated overnight at room temperature with one of the following primary antibodies: anti-chymase rabbit polyclonal antibody (1:1000 in PBS), anti-tryptase rabbit polyclonal antibody (1:1000 in PBS), or anti-glial fibrillary acidic protein (GFAP) rabbit polyclonal antibody (1:100 in PBS) (Santa Cruz, DBA, Milan, Italy). Sections were then washed with PBS and incubated with a biotin-conjugated goat anti-rabbit IgG and avidin–biotin–peroxidase complex (Vector Laboratories, DBA). No immunostaining was observed when either the primary or secondary antibody was omitted. Images captured (*n* = 5 photos from each sample collected from all rats in each experimental group) were quantitatively assessed for a difference in immunoreactivity by computer-assisted color image analysis (Leica QWin V3, Cambridge, UK). All analyses were carried out by an investigator blinded to the treatment. The number of GFAP-positive cells was acquired in three defined brain regions: (i) a cortical infarct border zone (IBZ), defined by the middle of the tangent to the cortical lesion; (ii) an ipsilateral cortical remote zone (RZ), adjacent to the interhemispheric gap; and (iii) a control region within the contralateral hemisphere (CH) cortex equivalent to the location of the IBZ. The number of GFAP-positive cells was counted in three sections per animal and presented as the number of positive cells per high-power field.

#### Western Blot Analysis

Cytosolic extracts were prepared with slight modifications. Briefly, brain tissues from each rat were suspended in extraction buffer A containing 10 mM HEPES, 10 mM KCl, 0.1 mM EDTA, 0.1 mM EGTA, 1 mM dithiothreitol, 0.5 mM phenylmethylsulfonyl fluoride, 3 μg/ml pepstatin A, 2 μg/ml leupeptin, 15 μg/ml trypsin inhibitor, and 40 μM benzamidine; homogenized at the highest setting for 2 min, and centrifuged at 13,000*g* for 3 min at 4 °C. Supernatants that represented the cytosolic fraction were retained and stored at −80 °C until being used. Equivalent amounts of protein for each sample were loaded per lane and electrophoretically separated using 10 % denaturing polyacrylamide gel electrophoresis (PAGE) (SDS-PAGE). The filters were blocked with 1× Tris-buffered saline (pH 7.5) and 5 % (*w*/*v*) nonfat dried milk for 1 h at room temperature and subsequently probed with the following primary antibodies: rabbit polyclonal anti-GFAP (1:2000), anti-brain-derived neurotrophic factor (BDNF) (1:1000; Santa Cruz Biotechnology), or anti-glial cell line-derived neurotrophic factor (GDNF) (1:1000; Santa Cruz Biotechnology), in 1× PBS, 5 % (*w*/*v*) nonfat dried milk, and 0.1 % Tween-2 at 4 °C overnight. Membranes were incubated with peroxidase-conjugated bovine anti-mouse IgG secondary antibody or peroxidase-conjugated goat anti-rabbit IgG (1:2000; Jackson ImmunoResearch, West Grove, PA, USA) for 1 h at room temperature. To control for protein loading, the blots were stripped by agitation with 3 % glycine (pH 2) and blocked in 1× PBS/5 % (*w*/*v*) nonfat dried milk for 1 h at room temperature and then probed for 2 h at room temperature with anti-β-actin antibody (Sigma-Aldrich). Relative expression of the protein bands was quantified by densitometric scanning of the X-ray films with a GS-700 Imaging Densitometer (Bio-Rad Laboratories) and a computer program (Molecular Analyst, IBM) and standardized for densitometric analysis to β-actin levels.

#### Materials

Unless otherwise stated, all compounds were obtained from Sigma-Aldrich Company Ltd. (Milan, Italy). All other chemicals were of the highest commercial grade available. All stock solutions were prepared in non-pyrogenic saline (0.9 % NaCl; Baxter, Italy). Co-ultraPEALut was kindly supplied by the Epitech Group (Saccolongo (PD), Italy). The methodology underlying its preparation has been described previously [33].

### Assessment of Co-ultraPEALut in Stroke Patients

An observational study to evaluate the effects of co-ultraPEALut (Glialia®) administration in stroke patients was carried out between April 2013 and June 2014, involving 37 neuromotor rehabilitation facilities throughout Italy. The patients selected had experienced a first ischemic stroke, were clinically stabilized, and underwent rehabilitative therapy. Individuals with previous hospitalizations for stroke and with hemorrhagic stroke were excluded. Of the 267 patients initially enrolled, 250 completed the study. Seventeen patients were excluded from statistical evaluation for the following reasons: bilateral stroke (4), no stroke at first event (3), ischemic event occurred ≥18 months before the start of the study (7), and inadequate information about ischemic event (3). Eighty-four percent of patients were undergoing rehabilitative treatment in inpatient centers and the remaining 16 % on an outpatient basis. Table 3 summarizes the patient demographics, while the location and type of stroke injury are collated in Table 4.

**Table 3:** Patient demographics

Total	250
Male	132
Female	118
Age (years)	
Mean ± SD	71.4 ± 12.4
Range	31–100
Inpatient, % (*n*)	84 (210)
Outpatient, % (*n*)	16 (40)

**Table 4:** Location and type of stroke injury

	% of patients
Location	
Supratentorial	35.9
Subtentorial	10.5
Anterior circle	22.7
Posterior circle	6.4
Lacunar	8.6
Multiple location	15.9
Type	
Diffuse of injury	62.0
Internal capsule	26.6
External capsule	11.4

Therapy with Glialia® commenced at a median time of 18 days after the onset of the acute phase of stroke for inpatients and after a median time of 104 days for patients undergoing rehabilitation on an outpatient basis. Written informed consent was obtained from each patient or responsible family member prior to the beginning of the study. All patients were administered Glialia® (composed of co-ultramicronized 700 mg PEA and 70 mg luteolin, in microgranular form) sublingually, twice daily (every 12 h) for 60 days in association with the specific therapy (e.g., thrombolytics, aspirin, and anticoagulants) normally administered and/or with drugs prescribed for comorbidities, where present in the patient.

All patients underwent the following evaluations at baseline (T0) and after 30 days (T30) of treatment: (i) stroke severity by the Canadian Neurological Scale (CNS) [46], a tool used to assess and monitor the neurological status of patients with stroke; (ii) impairment of cognitive abilities by the Mini Mental State Examination (MMSE) [47] adjusted for age and educational level; (iii) degree of spasticity, by means of the Ashworth Scale; (iv) pain, by the Numeric Rating Scale (NRS), a scale of 0 to 10 points with 0 being no pain at all and 10 being the worst pain imaginable; and (v) independence in activities of daily living by the Barthel Index, which scales functional capacity in terms of patient self-care [48]. Evaluation of the degree of autonomy and/or dependence of the patient from help, both physical and verbal, in carrying out daily living activities was performed also at the 60th day, concurrently with treatment end. During the period of rehabilitation therapy, patients were subjected to routine blood chemistry and hematology analyses and monitored for the possible occurrence of adverse events.

### Statistical Analysis

#### Preclinical Data

Statistically significant differences in neurological score were evaluated by one-way ANOVA, followed by the Newman–Keuls multiple comparison test. Statistically significant differences in the volume of brain ischemic damage were evaluated using unpaired Student’s *t* test. The results were analyzed by one-way ANOVA followed by the Bonferroni post hoc test for multiple comparisons in neurological score. All values are expressed as mean ± SD. A *P* value of less than 0.05 was considered significant.

#### Clinical Data

To evaluate changes of means in time, the generalized linear mixed model (GLMM) with SAS 9.2 was used. Variables such as gender, age, and hospitalization type were included in the model as covariates. The Bonferroni–Holm correction for multiple comparisons was adopted for the CNS evaluation.

## Results

### Physiologic Parameters and rCBF

**Fig. 1:**
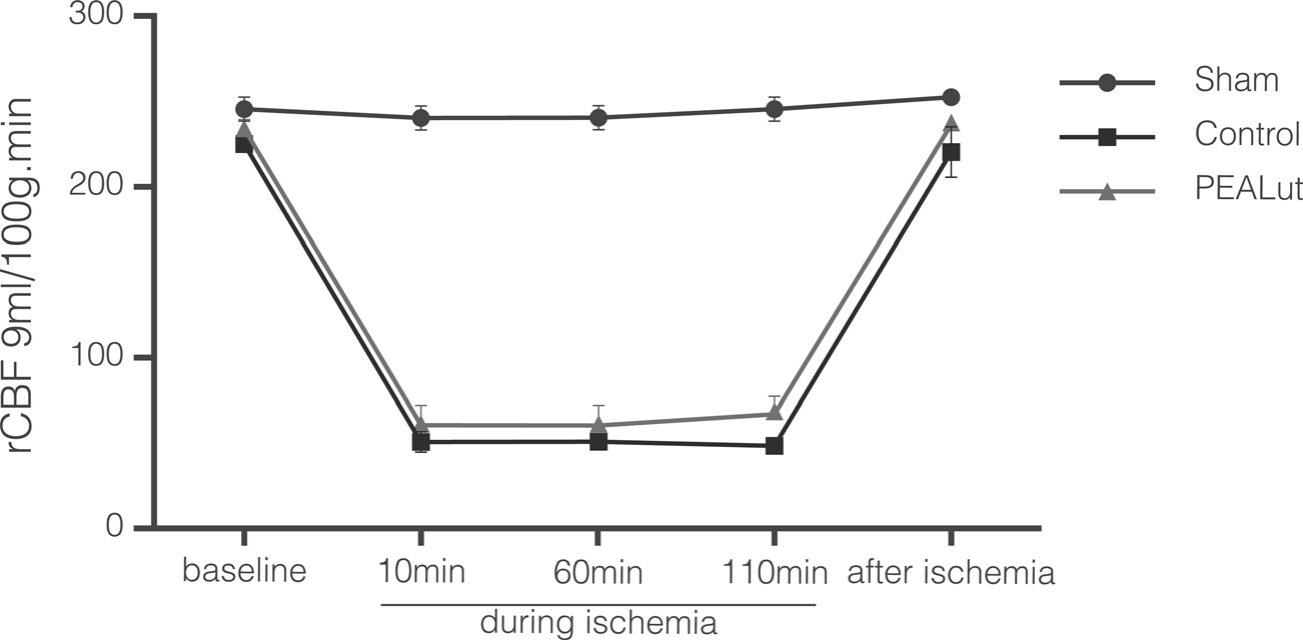
Regional cerebral blood flow analysis (*rCBF*). rCBF was monitored 10 min before ischemia (baseline); at 10, 60, and 110 min during ischemia; and 5 min after ischemia. rCBF monitoring was used to confirm successful induction of MCAo

The changes of physiological parameters are shown in Table 1. No statistical significance among different time points for any of these parameters was noted. All physiological parameters were within the normal range for the experimental process. There was no significant difference in rCBF between the control and treated group at the corresponding time points (Fig. 1). Monitoring of rCBF showed successful MCAo.

### Neuroprotective Effects of Co-ultraPEALut on Turning Behavior, Neurological Deficit, and Ischemic Brain Damage in MCAo Rats

Turning behavior after permanent intraluminal MCAo is a precocious index of neurological deficit and neuronal cell damage [40, 41]. One hour after MCAo, the rats engaged in turning behavior towards the side contralateral to the ischemic hemisphere. This acute behavioral response lasted several hours and was no longer evident 24 h after ischemia. Co-ultraPEALut, administered orally, blocked this acute behavioral response. Turning behavior, expressed as mean rotations per hour, was 788 ± 133 in MCAo vehicle-treated rats (*n* = 10) and 138 ± 42 (*n* = 10) in co-ultraPEALut-treated rats (mean ± SD). This effect of co-ultraPEALut was statistically significant (unpaired Student’s *t* test: *p* < 0.001 vs. vehicle-treated rats). Sham-operated rats did not show any turning behavior.

Neurological deficit and ischemic striatal and cortical damage of MCAo vehicle-treated, MCAo co-ultraPEALut-treated, and sham-operated rats were evaluated after 24 h (Table 2). Co-ultraPEALut significantly improved MCAo-induced neurological deficit in comparison to vehicle-treated rats and reduced both cortical and striatal damages in comparison to vehicle-treated rats. No damage was found in the sham-operated rats or in the hemisphere contralateral to the ischemic side.

### Effect of Co-ultraPEALut on Infarct Area and Morphological Changes

**Fig. 2:**
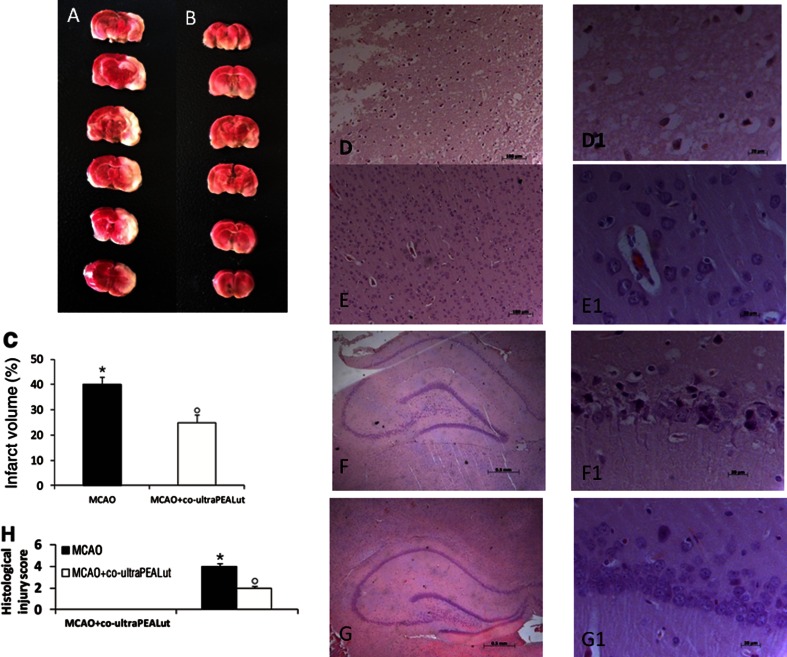
Co-ultraPEALut reduces infarct area and histological damage in a rat MCAo model. Co-ultraPEALut (1 mg/kg) was administered orally 1 h after ischemia and 6 h post-reperfusion. TTC staining of coronal brain sections 24 h after MCAo. Infarcted brain tissue appears unstained (**a**), while co-ultraPEALut treatment (**b**) significantly reduced infarct area (**c**). H&E staining of MCAo tissue shows a loss of neurons and the presence of multiple vacuolated interspaces (**d**, *D1*), which were significantly ameliorated in co-ultraPEALut-treated animals (**e**, *E1*). Moreover, damage to CA1 hippocampal neurons following ischemia/reperfusion (**f**, *F1*) was significantly attenuated by co-ultraPEALut (**g**, *G1*). This figure is representative of at least three independent experiments. Quantification of histological score (**h**). *p < 0.01, versus sham group; °*p* < 0.01, versus MCAo + vehicle group. ND: not detectable

Twenty-two hours after ischemia/reperfusion, the rats developed infarcts affecting the cortex and striatum (Fig. 2a). The co-ultraPEALut treatment group (*n* = 10) (Fig. 2b) had a significantly smaller infarct area (*n* = 10) (Fig. 2c) 24 h post-MCAo compared with the control (*n* = 10) (Fig. 2a). H&E staining revealed morphologically healthy cerebral neurons in the sham group (data not shown), whereas 24 h after reperfusion, the brain sections from ischemic rats showed a paucity of intact neurons in those areas (Fig. 2d (D1); see histological score H) and the presence of multiple vacuolated interspaces (Fig. 2d (D1); see histological score H). In contrast, the corresponding areas in the co-ultraPEALut group displayed partial neuronal cell loss only and the presence of intact neurons between the vacuolated spaces (Fig. 2e (E1); see histological score H). Hippocampal CA1 pyramidal cells 24 h after reperfusion were layered and arranged uniformly with large, round, transparent, and intact nuclei (data not shown). In the ischemia/reperfusion group, CA1 neurons were significantly reduced in numbers and characterized by pyknotic and indistinct nuclei (Fig. 2f (F1); see histological score H). Co-ultraPEALut treatment significantly decreased neuronal cell death in CA1 (Fig. 2g (G1); see histological score H).

### Effect of Co-ultraPEALut on Astrocyte Activation After MCAo

**Fig. 3:**
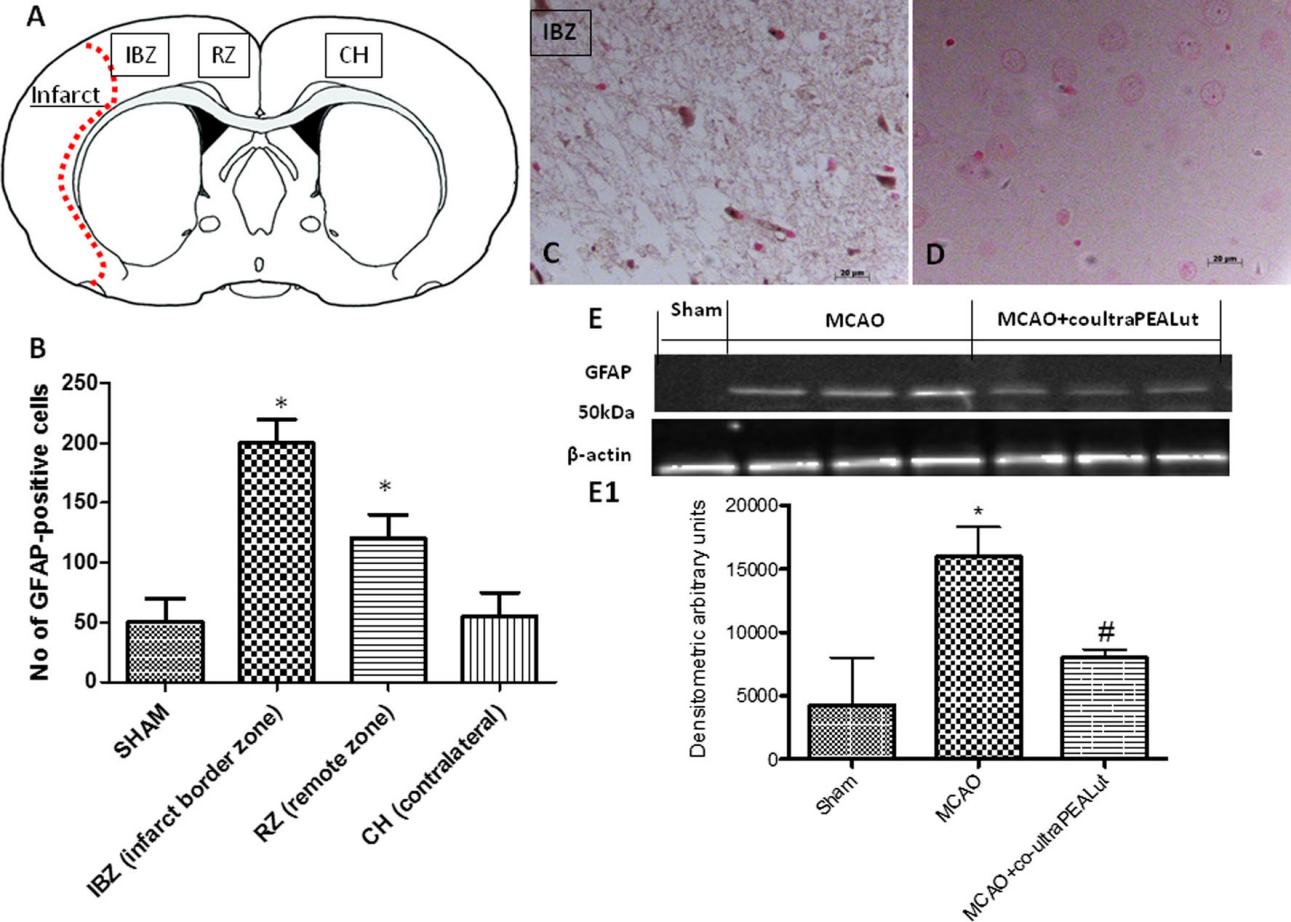
Co-ultraPEALut moderates MCAo-induced activation of astrocytes. A representative brain section showing the regions of interest used for analysis: cortical infarct border zone (*IBZ*), ipsilateral cortical remote zone (*RZ*), and contralateral hemisphere (*CH*) (**a**). The number of glial fibrillary acidic protein (*GFAP*)-positive cells was counted in three sections per animal in each experimental region and presented as the number of positive cells per high-power field (**b**). Immunohistochemical localization of GFAP shows positive staining in the IBZ of vehicle-treated MCAo animals (**c**) in contrast to the co-ultraPEALut-treated ischemic rats (**d**). Western blot analysis demonstrated a marked increase in GFAP-immunoreactive protein in ischemic tissue and its moderation by co-ultraPEALut treatment (**e**, *E1*). Values in *E1* are mean ± SD for five to six rats from each group. *p < 0.01, versus sham group; ^**#**^ *p* < 0.01, versus MCAo group. Data are representative of three independent experiments

Astrocytes were revealed by GFAP immunohistochemistry; active astrocytes were defined as GFAP-stained cells with an increased GFAP immunoreactivity. A basal number of astrocytes were detected in the sham controls (Fig. 3b). Twenty-two hours of ischemia/reperfusion resulted in significant increases in the number of astrocytes and GFAP immunoreactivity in the cortical IBZ and RZ when compared to the sham control or contralateral hemisphere (Fig. 3b, c). GFAP-positive astrocytes were more numerous in the border zone compared to the remote zone (Fig. 3b). The number of GFAP-stained cells in the contralateral hemisphere was similar to the sham control (Fig. 3c). Brain sections from sham-operated rats did not exhibit appreciable immunostaining for GFAP (data not shown), unlike infarct border zone sections from MCAo rats (Fig. 3c). Co-ultraPEALut reduced GFAP immunostaining in the infarct border zone of rats subjected to MCAo (Fig. 3d). Western blot analysis confirmed the immunohistochemical findings, with a low level of GFAP expression in extracts from the occluded hemisphere of sham-operated rats and a significant increase in the MCAo vehicle-treated group (Fig. 3e (E1)). Co-ultraPEALut decreased the damage-induced rise in GFAP expression to a significant extent (Fig. 3d (D1)).

### Co-ultraPEALut Limits Ischemia-Induced Loss of BDNF and GDNF Expression

**Fig. 4:**
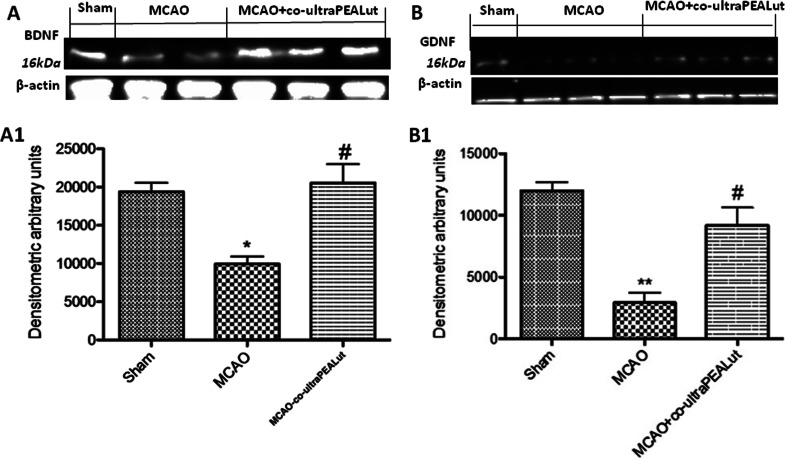
Co-ultraPEALut restores the MCAo-induced loss of BDNF and GDNF expression. Co-ultraPEALut (1 mg/kg) was administered orally 1 h after ischemia and 6 h post-reperfusion. Animals were sacrificed 24 h after MCAo, and brain tissue lysates were analyzed by Western blot for BDNF and GDNF. Ischemic rats exhibited a marked reduction in the levels of both BDNF (**a**, *A1*) and GDNF (**b**, *B1*) which were restored by co-ultraPEALut treatment. Data are mean ± SD for five to six rats from each group. *p < 0.01, versus sham group; **p < 0.001, versus sham group; ^#^ *p* < 0.01, versus MCAo group. Representative blots are shown in **a**, **b**, with quantification of all animals given in *A1*, *B1*

Brain tissues from MCAo rats showed a marked reduction in the levels of the neurotrophic factors BDNF and GDNF, as determined by Western blot 24 h post-ischemia (Fig. 4a (A1), b (B1)). Treatment with co-ultraPEALut increased the expression levels of both BDNF and GDNF in comparison to with non-treated injured rats.

### Co-ultraPEALut Reduces Ischemia-Induced Mast Cell Infiltration and Chymase and Tryptase Expression

**Fig. 5:**
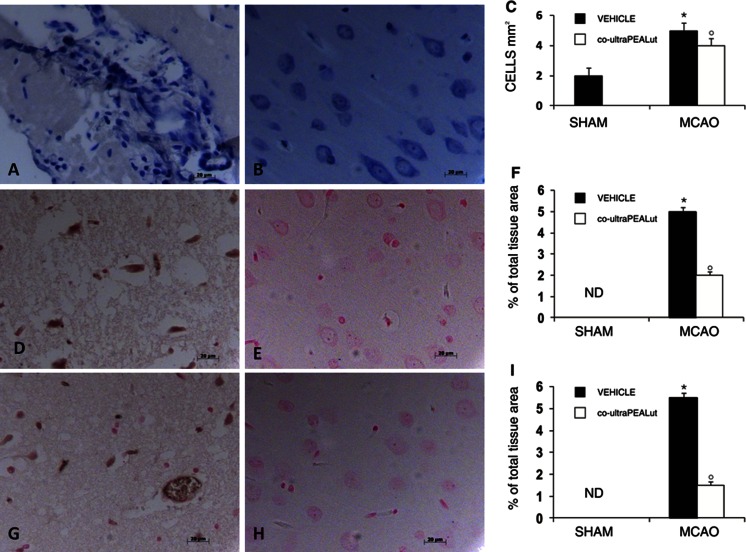
Effect of co-ultraPEALut treatment on mast cells in MCAo rats. Co-ultraPEALut (1 mg/kg) was administered orally 1 h after ischemia and 6 h post-reperfusion. Animals were sacrificed 24 h after MCAo, and tissues were prepared for immunohistochemical analysis. (**a**) Toluidine blue-stained mast cells with their characteristic metachromatic granular inclusions were evident in MCAo-vehicle treated rats. (**b**) Co-ultraPEALut-treated ischemic animals displayed only occasional cells of this type. (**c**) Mast cell density per unit area. A marked increase in immunoreactivity for the mast cell enzymes chymase (**d**) and tryptase (**g**) was seen in the brain sections from MCAo vehicle-treated rats. This expression was significantly attenuated in the MCAo group treated with co-ultraPEALut (**e**, **h**). Densitometric analysis for chymase (**f**) and tryptase (**i**) immunoreactivities is given (five photomicrographs from each sample collected from all rats in each experimental group) and was carried out using Optilab Graftek software on a Macintosh personal computer (CPU G3-266). Data, expressed as percentage of total tissue area, are mean ± SD. *p < 0.01, versus sham group; °*p* < 0.01, versus MCAo + vehicle group. *ND* not detectable

Toluidine blue staining of the brain tissue from MCAo vehicle-treated rats identified cells with metachromatic granules, a characteristic of mast cells (Fig. 5a, c). In contrast, significantly fewer cells of this type were seen in the ischemic tissue of rats treated with co-ultraPEALut (Fig. 5b, c) and none in sham-operated rats (data not shown). While immunoreactivity for chymase and tryptase, two serine peptidases characteristic of mast cells, was absent in brain tissues from sham-operated rats (data not shown), there were substantial increases 24 h after ischemia (Fig. 5d, g, respectively, see densitometries F and I). Chymase and tryptase expression was attenuated in tissue sections from rats receiving co-ultraPEALut (Fig. 5e, h, respectively, see densitometries F and I).

### Co-ultraPEALut Effect on Bax and Bcl-2 Expression

**Fig. 6:**
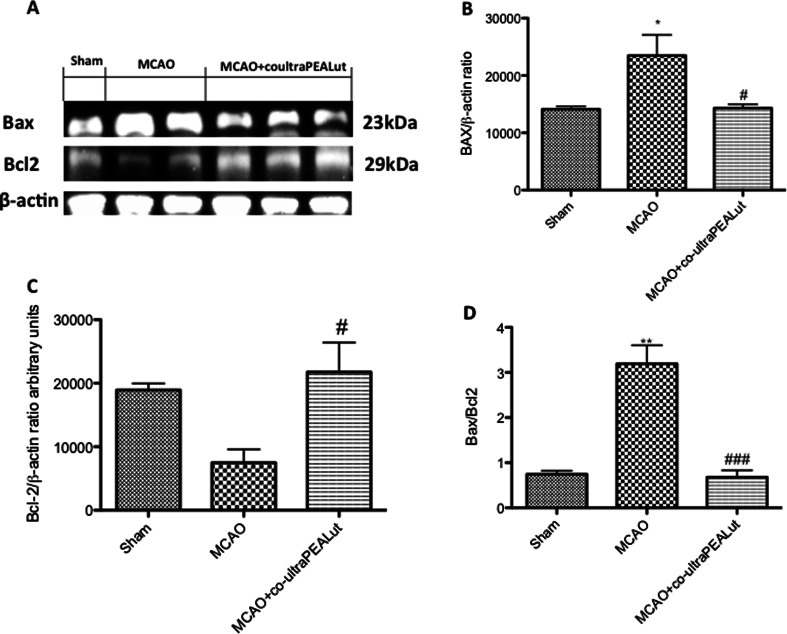
Effects of co-ultraPEALut on Bax and Bcl-2. Co-ultraPEALut (1 mg/kg) was administered orally 1 h after ischemia and 6 h post-reperfusion. Animals were sacrificed 24 h after MCAo, and brain tissue lysates were analyzed by Western blot for Bax and Bcl-2. (**a**, **b**) Co-ultraPEALut treatment reduced the levels of Bax in the brain of ischemic rats. β-Actin was used as internal control. (**a**, **c**) Co-ultraPEALut administration increased the Bcl-2 levels in the brain tissue of ischemic rats. Co-ultraPEALut administration normalized Bax/Bcl-2 expression levels (**d**). Data are mean ± SD for five to six rats from each group. *p < 0.01, versus sham group; **p < 0.001, versus sham group; ^###^ *p* < 0.001; ^#^ *p* < 0.01, versus MCAo group. Representative blots are shown in **a**, with quantification of all animals given in **b**–**d**

Western blot analysis was employed to determine the levels of Bax and Bcl-2 expression. As shown in Fig. 6, co-ultraPEALut administration effectively normalized the Bax level in the brain of rats subjected to MCAo (Fig. 6b). At the same time, co-ultraPEALut significantly restored the reduction in Bcl-2 expression observed in MCAo rats (Fig. 6c). As such, co-ultraPEALut administration normalized the Bax/Bcl-2 expression level (Fig. 6d).

### Clinical Assessment of Stroke Patients Treated with Glialia®

**Fig. 7:**
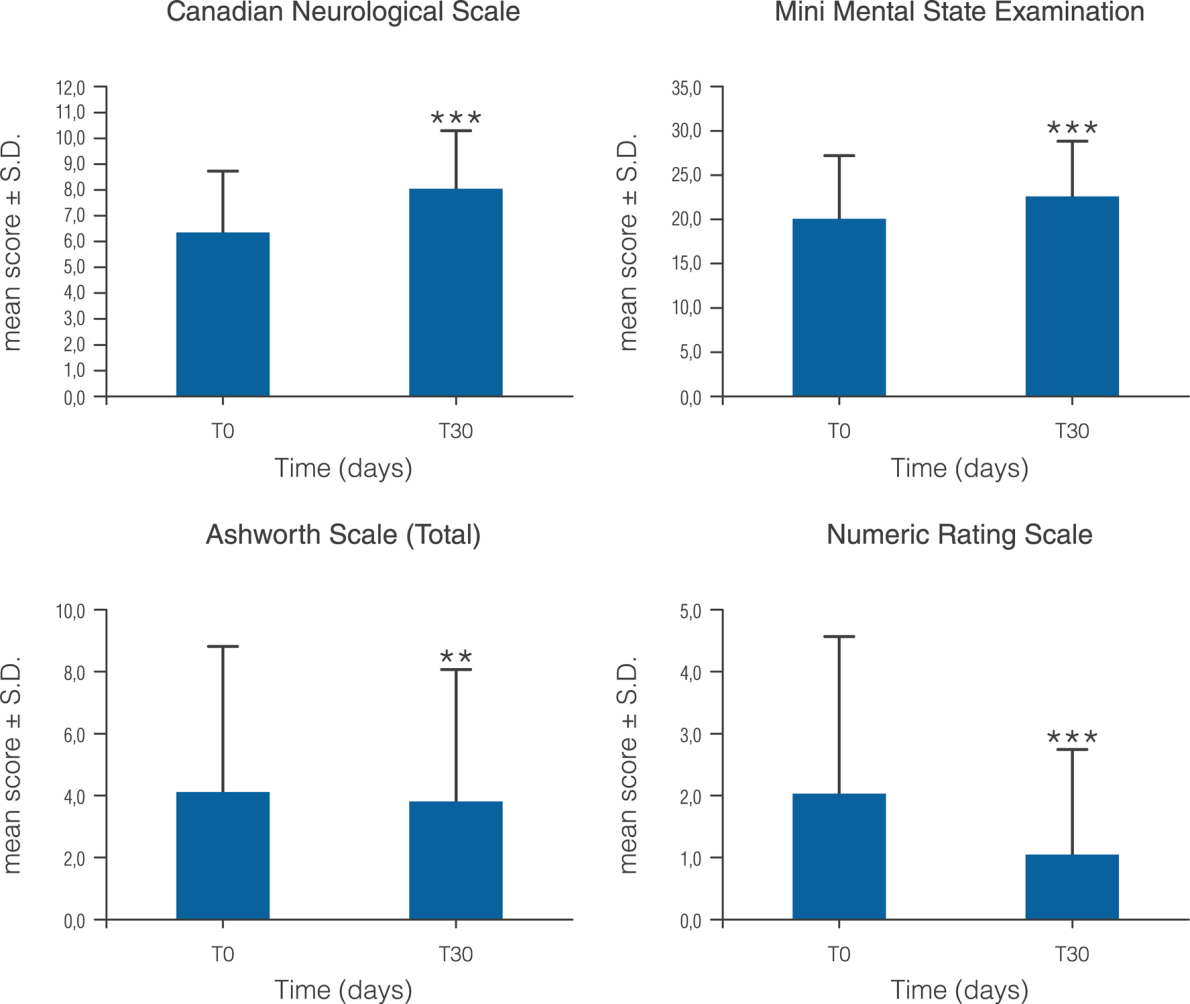
Neurological scoring for stroke patients treated with Glialia®. Patients were administered Glialia®, as described in the “Materials and Methods” section, for a period of 60 days and underwent the following evaluations at baseline (T0) and after 30 days (T30). *Canadian Neurological Score* values were 6.4 ± 0.16 and 8.1 ± 0.15 at T0 (242 patients) and T30 (237 patients), respectively. Delta: T30–T0 was calculated on the basis of 237 patients. There was a significant difference in time (***p < 0.0001) between T0 and T30. *Mini Mental State Exam* values were 20.2 ± 0.57 and 22.7 ± 0.47 at T0 (169 patients) and T30 (196 patients), respectively. Delta: T30–T0 was calculated on the basis of 167 patients. There were significant differences in time (***p < 0.0001) between T0 and T30. Seventy-two patients were unable to carry out the exam at T0; 28 of these were able to take the test at T30. *Ashworth Scale* values were 4.1 ± 0.32 and 3.7 ± 0.27 at T0 (246 patients) and T30 (244 patients), respectively. Delta: T30–T0 was calculated on the basis of 244 patients. There was a significant difference in time (**p < 0.0015) between T0 and T30. *Numerical Rating Scale* values were 2.1 ± 0.17 and 1.1 ± 0.11 at T0 (242 patients) and T30 (241 patients), respectively. Delta: T30–T0 was calculated on the basis of 241 patients. There was a significant difference in time (***p < 0.0001)

Nine of the 250 enrolled patients dropped out because of death owing to severity of concomitant diseases or seriousness of ischemic state (2), transfer to another rehabilitation center (3), onset of diarrhea (2), gastric discomfort (1), and agitation (1). None of these reasons were considered by the attending physician to be treatment related. The CNS showed a significant improvement over time (*p* < 0.0001), with the mean total score increasing by 1.7 ± 0.10 between T0 and T30 (Fig. 7). Furthermore, GLMM analysis showed that while baseline average score in women was indicative of greater severity than males (*p* = 0.0049), improvement over time was independent of both gender and recovery type.

Cognitive function evaluation performed by MMSE showed a significant improvement (*p* < 0.0001) compared to baseline performance after 30 days of Glialia® treatment (Fig. 7). The average score over these 30 days of treatment increased from 20.2 ± 0.57 (T0) to 22.7 ± 0.47 (T30). Out of the 78 patients who were unable to perform the MMSE at baseline, 28 were capable of carrying out the test at follow-up after 30 days of treatment. The MMSE analysis showed also that at baseline, female patients had a significantly (*p* = 0.0450) greater cognitive impairment compared to males. However, there was no time × recovery and gender effect.

Muscle spasticity evaluated by the Ashworth Scale showed that the global spasticity significantly improved over time (*p* < 0.0015) (Fig. 7). The severity of spasticity at baseline was greater in outpatients (*p* < 0.0003) than in inpatients, and outpatient improvement was statistically significantly greater (*p* < 0.0075) than that of inpatients. These observations apply to the assessment of spasticity both overall and at the level of each limb.

The average pain intensity detected by NRS fell within a range of values considered clinically very mild. The average value for NRS at baseline was 2.1 ± 0.17; after 30 days of Glialia® treatment, this had decreased one point to a mean value of 1.1 ± 0.11 (*p* < 0.0001) (Fig. 7). The baseline mean score of pain intensity appeared higher in outpatients (3.1 ± 0.49) compared to inpatients (1.9 ± 0.18) and reached a statistical significance (*p* = 0.0014).

**Fig. 8:**
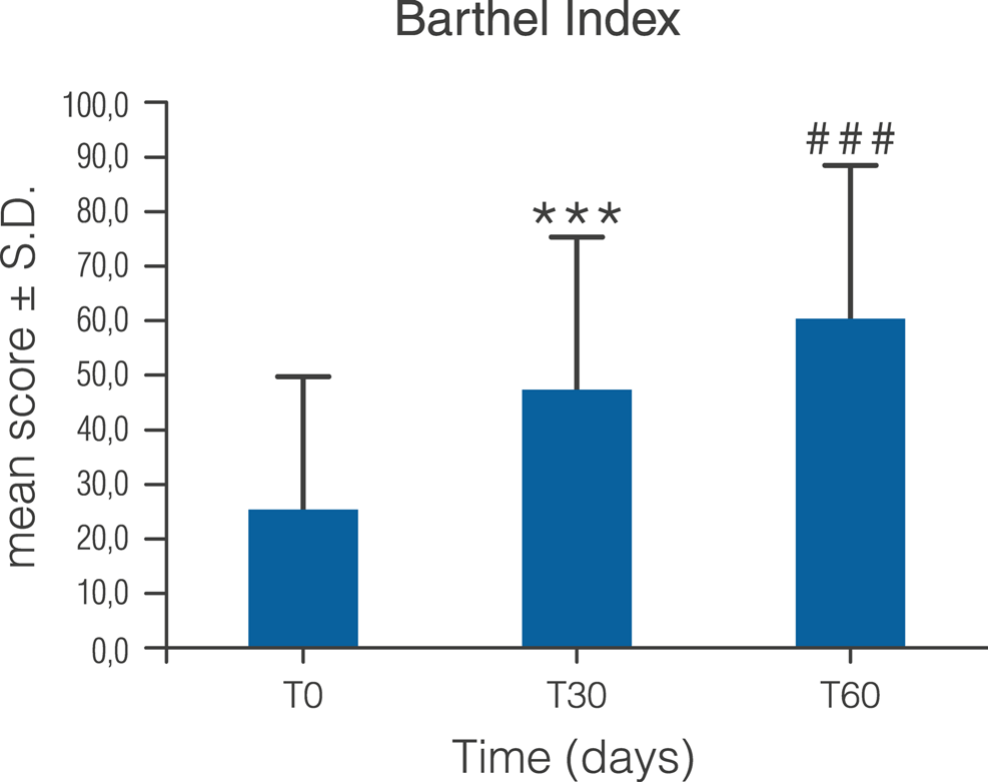
Barthel Index score for stroke patients treated with Glialia®. Patients were administered Glialia®, as described in the “Materials and Methods” section, for a period of 60 days. Barthel Index values were 26.6 ± 1.69, 48.3 ± 1.91, and 60.5 ± 1.95 at T0 (242 patients), T30 (229 patients), and T60 (218 patients), respectively. There was a significant difference in the improvement between T0 and T30 (***p < 0.0001) and between T0 and T60 (^###^ *p* < 0.0001). Moreover, there was a highly significant difference also between T30 and T60 (*p* < 0.0001). Female patients exhibited lower scores than males, and disability was worse in inpatients

Patients’ independence and mobility in daily living activities (Barthel Index) showed a significant improvement (*p* < 0.0001) after 30 and 60 days of treatment (mean scores of 26.6 ± 1.69, 48.3 ± 1.91, and 60.5 ± 1.95 at T0, T30, and T60, respectively) (Fig. 8). Moreover, there was a highly significant difference between T30 and T60, indicative of a continuing improvement with time. The baseline degree of disability in daily living activity was significantly more severe in inpatients (*p* < 0.0003) than outpatients and in females (*p* < 0.0047) compared to males; improvement over time, however, appeared better in inpatients and was not influenced by gender.

Tolerability to Glialia® treatment was excellent, with no adverse events ever having been observed over the course of this study. Further, routine blood chemistry and hematology analyses did not reveal any deviations from their normal ranges in relation to Glialia® treatment.

## Discussion

Cerebral ischemia continues to represent one of the principal unmet medical needs in today’s society. Stroke is especially devastating, given that it constitutes the most frequent cause of neurological disability worldwide. The underlying cellular mechanisms of stroke neuropathology are complex. An initial episode of focal hypoperfusion subsequently leads to excitotoxicity, oxidative damage, microvascular injury, blood–brain barrier dysfunction, and post-ischemic inflammation [4, 49–51]. Despite almost four decades of experimental animal investigations on stroke and the identification of a spectrum of anti-inflammatory/neuroprotective compounds [11–15], translatability of these findings to human clinical trials until now have been proven uniformly disappointing [3, 16]. In the present study, we describe the neuroprotective effects of co-ultramicronized palmitoylethanolamide/luteolin in a rat model of focal cerebral ischemia and, more importantly, the ability of co-ultraPEALut (Glialia®) to improve the neurological status of stroke patients undergoing neurorehabilitation.

Focal cerebral ischemia is accompanied by reactive astrogliosis [52] and activation of microglial cells in the hippocampal area [53]. Reactive gliosis can lead to the production of excessive amounts of cytokines as well as inflammatory products that exacerbate ischemic damage [54]. In our study, an increased in GFAP immunoreactivity was observed in the MCAo group in comparison to sham-operated animals while there was a marked reduction in the co-ultraPEALut-treated group. As previously reported [55], co-ultraPEALut is able to significantly reduce expression levels of GFAP after MCAo. In the current MCAo model, oral administration of co-ultraPEALut 1 h after ischemia and 6 h after reperfusion improved neurological score, reduced lesion size and histological damage, inhibited mast cell infiltration/degranulation and astrocyte activation (as measured by GFAP accumulation, a characteristic neuropathologic feature of ischemic brain injury [56]), and restored expression of BDNF and GDNF. Experimental and clinical studies have shown that BDNF and GDNF are upregulated at very early stages during brain ischemia [56]. Furthermore, exogenous administration of GDNF and BDNF reduced the toxic effects of excitatory amino acids, attenuated nitric oxide production, and lowered apoptosis/cell death in stroke animal models [56, 57]. In our preclinical findings, protein analysis showed that both BDNF and GFAP levels were downregulated by cerebral ischemia while a local and sustained increase in their expression in the perilesioned tissue followed oral administration of co-ultraPEALut.

Mast cell activation and degranulation is known to contribute to blood–brain barrier disruption in cerebral ischemia [58]. Other elements play a role in ischemic brain damage, such as activation of the transcription factor nuclear factor-κB [59, 60], inducible nitric oxide synthase [61, 62], and poly(ADP-ribose)synthetase [63]; increased expression of the pro-apoptotic protein Bax [64]; and decreased expression of the anti-apoptotic molecule Bcl-2 [65]. In the present study, co-ultraPEALut significantly reduced nuclear factor-κB translocation, attenuated poly(ADP-ribose)synthetase activation, and normalized Bax/Bcl-2 expression levels. These results are qualitatively similar to our earlier investigations with PEA alone, although in the latter, 10-fold higher doses of PEA (compared to co-ultraPEALut) were required for efficacy [55, 66].

PEA actions are mediated, at least in part, by activation of peroxisome proliferator-activated receptors, accompanied by a decrease in neutrophil influx and expression of pro-inflammatory proteins, such as inducible nitric oxide synthase and cyclooxygenase-2 [39, 67]. Luteolin displays specific anti-inflammatory effects, which are only partly explained by its antioxidant capacities. The anti-inflammatory activity of luteolin includes activation of antioxidative enzymes, suppression of the nuclear factor-κB pathway, and inhibition of pro-inflammatory substances [68, 69]. The molecular basis behind the superior pharmacological efficacy of co-ultraPEALut compared to comparable concentrations of the single chemical components is currently under investigation.

Although inflammatory signaling is considered to participate in the early post-ischemic period, as the ischemic cascade progresses, cell death leads to a new phase of the inflammatory response, whereby the immune system becomes activated. There is evidence that both microglia and mast cells have a role at later times following the ischemic episode. Mast cells possess a potent armamentarium to target the components of the blood–brain barrier and basal lamina shortly after their activation in ischemia, whereas de novo production of mediators reactivates and maintains the process over the longer term [70–73]. Further, a newly published study demonstrates that mast cells can undergo a delayed and long-term activation following traumatic brain injury [74]. While the latter condition is not stroke, this report demonstrates that mast cells are not only early responders but also long-term players in brain injury. Further, it bears keeping in mind that in terms of *acute* versus *late-stage* treatment regimens in rats and man, one cannot necessarily make a direct comparison between time scales. In other words, a patient treated starting 60 days post-stroke does not have a rat equivalent.

Despite literally hundreds of compounds and interventions that provide benefit in experimental models of cerebral ischemia, efficacy in humans remains to be demonstrated [75]. Many patients with ischemic stroke, despite optimal medical treatment received during the acute phase, often fail to recover (or only partially), leading to persistent disability requiring rehabilitation. As Glialia® is already a marketed product, we investigated whether treatment with Glialia®, carried out simultaneously with rehabilitation therapy, can bring about a better functional recovery in stroke patients in the subacute phase. The observations reported here demonstrate that a positive outcome in an animal stroke model can be translated into stroke patients. Open studies, however, necessarily impose certain limitations as, for example, the lack of a randomized controlled trial’s robustness and the absence of a control group. Further, outcome of an observational study may be biased by patient attributes that affect treatment selection. Formal clinical trials typically employ randomization to address some of these issues by balancing baseline characteristics among the treatment groups. This open study was intended to consider patients who are observed in the context of neurological disability resulting from cerebral ischemia and undergoing rehabilitation—independent of their gravity. The large patient base provided a range in times from initial ischemic episode until the beginning of treatment with Glialia® and can serve to gauge the evolution of a patient’s disability with treatment in a long-term rehabilitation setting with the same rehabilitation team. We believe that such information may allow one to derive considerations for clinical practice on choice of therapy to combine with rehabilitation for patients with persistent stroke-related neurological disabilities.

Systematic reviews comparing the results of randomized controlled trials and observational studies of the same agents have failed to demonstrate significant differences in outcomes across multiple study designs [76]. In order to address the absence of a control patient group, we compared the results obtained by the CNS, Ashworth Scale, and Barthel Index in patients treated with Glialia® with historical literature [77] data observed in patients having similar pathologic conditions but never receiving Glialia® (Table 5). A *z* test comparing the mean value reported (standard error was not given) and that of the present study was performed; this comparison is limited to the observed mean value and a fixed value. As such, the test tends to overestimate the probability value and therefore was fixed (alpha = 0.01).

**Table 5:** Comparison of current multicenter study results with reported values for stroke patients not receiving Glialia®

	Multicenter study	Study A^a^	Study B^b^
	Δ^c^	Δ^c^	Δ^c^
Canadian Neurological Scale	1.80	0.01 (*p* < 0.0001)	1.02 (*p* < 0.0001)
Ashworth Scale	−0.20	–	0.58 (*p* < 0.0009)
Barthel Index	36.2	0.62 (*p* < 0.0001)	34.8 n.s.

^a^Fornasari et al. [77]

^b^Current data (C. Cisari)

^c^The mean value achieved between basal and study end

These caveats aside, this represents the first description of co-ultraPEALut administration to human stroke patients and clinical improvement not otherwise expected from spontaneous recovery. Based on these observations, we believe that controlled trials are warranted to confirm the utility of co-ultraPEALut to improve clinical outcome in human stroke, also in consideration of the excellent tolerability associated with Glialia® treatment. A double-blind, randomized, and placebo-controlled trial of Glialia® in stroke patients within 12 h of the initial ischemic episode has been initiated.

## References

[1] Thrift AG, Cadilhac DA, Thayabaranathan T, Howard G, Howard VJ, Rothwell PM (2014). Global stroke statistics. Int J Stroke.

[2] American Heart Association Statistics Committee and Stroke Statistics Committee (2014). Heart disease and stroke statistics—2014 update: a report from the American Heart Association. Circulation.

[3] Corbett D, Jeffers M, Nguemeni C, Gomez-Smith M, Livingston-Thomas J (2015). Lost in translation: rethinking approaches to stroke recovery. Prog Brain Res.

[4] Wang Q, Tang XN, Yenari MA (2007). The inflammatory response in stroke. J Neuroimmunol.

[5] Kawabori M, Yenari MA (2015). Inflammatory responses in brain ischemia. Curr Med Chem.

[6] Chew LJ, Takanohashi A, Bell M (2006). Microglia and inflammation: impact on developmental brain injuries. Ment Retard Dev Disabil Res Rev.

[7] Hanisch U-K, Kettenmann H (2007). Microglia: active sensor and versatile effector cells in the normal and pathologic brain. Nat Neurosci.

[8] Jin Y, Silverman AJ, Vannucci SJ (2009). Mast cells are early responders after hypoxia-ischemia in immature rat brain. Stroke.

[9] Silver R, Curley JP (2013). Mast cells on the mind: new insights and opportunities. Trends Neurosci.

[10] Sofroniew MV (2010). Astrocytes: biology and pathology. Acta Neuropath.

[11] Calabresi P, Cupini LM, Centonze D, Pisani F, Bernardi G (2003). Antiepileptic drugs as a possible neuroprotective strategy in brain ischemia. Ann Neurol.

[12] ArunaDevi R, Lata S, Bhadoria BK, Ramteke VD, Kumar S, Sankar P (2010). Neuroprotective effect of 5,7,3′,4′,5′-pentahydroxy dihydroflavanol-3-O-(2″-O-galloyl)-beta-D-glucopyranoside, a polyphenolic compound in focal cerebral ischemia in rat. Eur J Pharmacol.

[13] ArunaDevi R, Ramteke VD, Kumar S, Shukla MK, Jaganathan S, Kumar D (2010). Neuroprotective effect of s-methylisothiourea in transient focal cerebral ischemia in rat. Nitric Oxide.

[14] Ye Q, Li Q, Zhou Y, Xu L, Mao W, Gao Y (2015). Synthesis and evaluation of 3-(furo[2,3-b]pyridin-3-yl)-4-(1H-indol-3-yl)-maleimides as novel GSK-3β inhibitors and anti-ischemic agents. Chem Biol Drug Des.

[15] Jin Z, Liang J, Wang J, Kolattukudy PE (2015). MCP-induced protein 1 mediates the minocycline-induced neuroprotection against cerebral ischemia/reperfusion injury in vitro and in vivo. J Neuroinflammation.

[16] Sughrue ME, Grobelny BT, Ducruet AF, Komotar RJ, Mocco J, Sciacca RR (2010). Data presentation in rodent stroke studies and the predictive value of confidence intervals. J Clin Neurosci.

[17] Hussain MS, Shuaib A (2008). Research into neuroprotection must continue … but with a different approach. Stroke.

[18] Prestigiacomo CJ, Kim SC, Connolly ES, Liao H, Yan SF, Pinsky DJ (1999). CD18-mediated neutrophil recruitment contributes to the pathogenesis of reperfused but not nonreperfused stroke. Stroke.

[19] Zhang L, Zhang ZG, Zhang RL, Lu M, Krams M, Chopp M (2003). Effects of a selective CD11b/CD18 antagonist and recombinant human tissue plasminogen activator treatment alone and in combination in a rat embolic model of stroke. Stroke.

[20] Enlimomab Acute Stroke Trial Investigators (2001). Use of anti-ICAM-1 therapy in ischemic stroke: results of the Enlimomab Acute Stroke Trial. Neurology.

[21] Becker KJ (2002). Anti-leukocyte antibodies: LeukArrest (Hu23F2G) and enlimomab (R6.5) in acute stroke. Curr Med Res Opin.

[22] Buckley CD, Gilroy DW, Serhan CN, Stockinger B, Tak PP (2013). The resolution of inflammation. Nat Rev Immunol.

[23] Pacher P, Bátkai S, Kunos G (2006). The endocannabinoid system as an emerging target of pharmacotherapy. Pharmacol Rev.

[24] Lambert DM, Vandevoorde S, Jonsson KO, Fowler CJ (2002). The palmitoylethanolamide family: a new class of anti-inflammatory agents?. Curr Med Chem.

[25] Skaper SD, Facci L (2012). Mast cell-glia axis in neuroinflammation and therapeutic potential of the anandamide congener palmitoylethanolamide. Philos Trans R Soc Lond B Biol Sci.

[26] Skaper SD, Facci GP (2014). Mast cells, glia and neuroinflammation: partners in crime?. Immunology.

[27] Impellizzeri D, Esposito E, Attley J, Cuzzocrea S (2014). Targeting inflammation: new therapeutic approaches in chronic kidney disease (CKD). Pharmacol Res.

[28] Alhouayek M, Muccioli GG (2014). Harnessing the anti-inflammatory potential of palmitoylethanolamide. Drug Discov Today.

[29] Esposito E, Cordaro M, Cuzzocrea S (2014). Roles of fatty acid ethanolamides (FAE) in traumatic and ischemic brain injury. Pharmacol Res.

[30] Fidaleo M, Fanelli F, Ceru MP, Moreno S (2014). Neuroprotective properties of peroxisome proliferator-activated receptor alpha (PPARα) and its lipid ligands. Curr Med Chem.

[31] Crupi R, Paterniti I, Ahmad A, Campolo M, Esposito E, Cuzzocrea S (2013). Effects of palmitoylethanolamide and luteolin in an animal model of anxiety/depression. CNS Neurol Disord: Drug Targets.

[32] Paterniti I, Cordaro M, Campolo M, Siracusa R, Cornelius C, Navarra M (2014). Neuroprotection by association of palmitoylethanolamide with luteolin in experimental Alzheimer’s disease models: the control of neuroinflammation. CNS Neurol Disord: Drug Targets.

[33] Paterniti I, Impellizzeri D, Di Paola R, Navarra M, Cuzzocrea S, Esposito E (2013). A new co-ultramicronized composite including palmitoylethanolamide and luteolin to prevent neuroinflammation in spinal cord injury. J Neuroinflammation.

[34] Cordaro M, Impellizzeri D, Paterniti I, Bruschetta G, Siracusa R, De Stefano D (2015). Neuroprotective effects of Co-ultraPEALut on secondary inflammatory process and autophagy involved in traumatic brain injury. J Neurotrauma.

[35] Longa EZ, Weinstein PR, Carlson S, Cummins R (1989). Reversible middle cerebral artery occlusion without craniectomy in rats. Stroke.

[36] Melani A, Pantoni L, Corsi C, Bianchi L, Monopoli A, Bertorelli R (1999). Striatal outflow of adenosine, excitatory amino acids, gamma-aminobutyric acid, and taurine in awake freely moving rats after middle cerebral artery occlusion: correlations with neurological deficit and histopathological damage. Stroke.

[37] Guo C, Yin Y, Duan J, Zhu Y, Yan J, Wei G (2015). Neuroprotective effect and underlying mechanism of sodium danshensu [3-(3,4-dihydroxyphenyl) lactic acid from Radix and Rhizoma Salviae miltiorrhizae = Danshen] against cerebral ischemia and reperfusion injury in rats. Phytomedicine.

[38] Lu XC, Massuda E, Lin Q, Li W, Li JH, Zhang J (2003). Post-treatment with a novel PARG inhibitor reduces infarct in cerebral ischemia in the rat. Brain Res.

[39] Impellizzeri D, Bruschetta G, Cordaro M, Crupi R, Siracusa R, Esposito E (2014). Micronized/ultramicronized palmitoylethanolamide displays superior oral efficacy compared to nonmicronized palmitoylethanolamide in a rat model of inflammatory pain. J Neuroinflammation.

[40] Melani A, Pantoni L, Bordoni F, Gianfriddo M, Bianchi L, Vannucchi MG (2003). The selective A2A receptor antagonist SCH 58261 reduces striatal transmitter outflow, turning behavior and ischemic brain damage induced by permanent focal ischemia in the rat. Brain Res.

[41] Garcia JH, Wagner S, Liu KF, Hu XJ (1995). Neurological deficit and extent of neuronal necrosis attributable to middle cerebral artery occlusion in rats. Statistical validation. Stroke.

[42] Barber PA, Hoyte L, Colbourne F, Buchan AM (2004). Temperature-regulated model of focal ischemia in the mouse: a study with histopathological and behavioral outcomes. Stroke.

[43] Schomacher M, Müller HD, Sommer C, Schwab S, Schäbitz WR (2008). Endocannabinoids mediate neuroprotection after transient focal cerebral ischemia. Brain Res.

[44] Hara H, Friedlander RM, Gagliardini V, Ayata C, Fink K, Huang Z (1997). Inhibition of interleukin 1beta converting enzyme family proteases reduces ischemic and excitotoxic neuronal damage. Proc Natl Acad Sci U S A.

[45] Schabitz WR, Li F, Irie K, Sandage BW, Locke KW, Fisher M (1999). Synergistic effects of a combination of low-dose basic fibroblast growth factor and citicoline after temporary experimental focal ischemia. Stroke.

[46] Côté R, Battista RN, Wolfson C, Boucher J, Adam J, Hachinski V (1989). The Canadian Neurological Scale: validation and reliability assessment. Neurology.

[47] Folstein MF, Folstein SE, McHugh PR (1975). “Mini-mental state”: a practical method for grading the cognitive state of patients for the clinician. J Psychiat Res.

[48] Shah S, Cooper B (1991). Documentation for measuring stroke rehabilitation outcomes. Aust Med Rec J.

[49] Trendelenburg G (2014). Molecular regulation of cell fate in cerebral ischemia: role of the inflammasome and connected pathways. J Cereb Blood Flow Metab.

[50] Lai TW, Zhang S, Wang YT (2014). Excitotoxicity and stroke: identifying novel targets for neuroprotection. Prog Neurobiol.

[51] Moretti R, Pansiot J, Bettati D, Strazielle N, Ghersi-Egea JF, Damante G (2015). Blood-brain barrier dysfunction in disorders of the developing brain. Front Neurosci.

[52] Burtrum D, Silverstein FS (1994). Hypoxic-ischemic brain injury stimulates glial fibrillary acidic protein mRNA and protein expression in neonatal rats. Exp Neurol.

[53] Gehrmann J, Banati RB, Wiessner C, Hossmann KA, Kreutzberg GW (1995). Reactive microglia in cerebral ischaemia: an early mediator of tissue damage?. Neuropathol Appl Neurobiol.

[54] Walker EJ, Rosenberg GA (2009). TIMP-3 and MMP-3 contribute to delayed inflammation and hippocampal neuronal death following global ischemia. Exp Neurol.

[55] Ahmad A, Genovese T, Impellizzeri D, Crupi R, Velardi E, Marino A (2012). Reduction of ischemic brain injury by administration of palmitoylethanolamide after transient middle cerebral artery occlusion in rats. Brain Res.

[56] Han BH, Holtzman DM (2000). BDNF protects the neonatal brain from hypoxic-ischemic injury in vivo via the ERK pathway. J Neurosci.

[57] Wang Y, Chang CF, Morales M, Chiang YH, Hoffer J (2002). Protective effects of glial cell line-derived neurotrophic factor in ischemic brain injury. Ann NY Acad Sci.

[58] Mattila OS, Strbian D, Saksi J, Pikkarainen TO, Rantanen V, Tatlisumak T (2011). Cerebral mast cells mediate blood-brain barrier disruption in acute experimental ischemic stroke through perivascular gelatinase activation. Stroke.

[59] Barnes PJ, Karin M (1997). Nuclear factor-kappaB: a pivotal transcription factor in chronic inflammatory diseases. N Engl J Med.

[60] Bowie A, O’Neill LA (2000). Oxidative stress and nuclear factor-kappaB activation: a reassessment of the evidence in the light of recent discoveries. Biochem Pharmacol.

[61] Love S (1999). Oxidative stress in brain ischemia. Brain Pathol.

[62] Lerouet D, Beray-Berthat V, Palmier B, Plotkine M, Margaill I (2002). Changes in oxidative stress, iNOS activity and neutrophil infiltration in severe transient focal cerebral ischemia in rats. Brain Res.

[63] Tokime T, Nozaki K, Sugino T, Kikuchi H, Hashimoto N, Ueda K (1998). Enhanced poly(ADP-ribosyl)ation after focal ischemia in rat brain. J Cereb Blood Flow Metab.

[64] Finucane DM, Bossy-Wetzel E, Waterhouse NJ, Cotter TG, Green DR (1999). Bax-induced caspase activation and apoptosis via cytochrome c release from mitochondria is inhibitable by Bcl-xL. J Biol Chem.

[65] Kowaltowski AJ, Fenton RG, Fiskum G (2004). Bcl-2 family proteins regulate mitochondrial reactive oxygen production and protect against oxidative stress. Free Radic Biol Med.

[66] Ahmad A, Crupi R, Impellizzeri D, Campolo M, Marino A, Esposito E (2012). Administration of palmitoylethanolamide (PEA) protects the neurovascular unit and reduces secondary injury after traumatic brain injury in mice. Brain Behav Immun.

[67] Citraro R, Russo E, Scicchitano F, van Rijn CM, Cosco D, Avagliano C (2013). Antiepileptic action of N-palmitoylethanolamine through CB1 and PPAR-α receptor activation in a genetic model of absence epilepsy. Neuropharmacology.

[68] Middleton E, Kandaswami C, Theoharides TC (2000). The effects of plant flavonoids on mammalian cells: implications for inflammation, heart disease, and cancer. Pharmacol Rev.

[69] Seelinger G, Merfort I, Schempp CM (2008). Anti-oxidant, anti-inflammatory and anti-allergic activities of luteolin. Planta Med.

[70] Lovatel GA, Bertoldi K, Elsnerb VR, Piazza FV, Basso CG, Moysés Fdos S (2014). Long-term effects of pre and post-ischemic exercise following global cerebral ischemia on astrocyte and microglia functions in hippocampus from Wistar rats. Brain Res.

[71] Yang Y, Salayandia VM, Thompson JF, Yang LY, Estrada EY, Yang Y (2015). Attenuation of acute stroke injury in rat brain by minocycline promotes blood-brain barrier remodeling and alternative microglia/macrophage activation during recovery. J Neuroinflammation.

[72] Brifault C, Gras M, Liot D, May V, Vaudry D, Wurtz O (2015). Delayed pituitary adenylate cyclase-activating polypeptide delivery after brain stroke improves functional recovery by inducing m2 microglia/macrophage polarization. Stroke.

[73] Strbian D, Kovanen PT, Karjalainen-Lindsberg ML, Tatlisumak T, Lindsberg PJ (2009). An emerging role of mast cells in cerebral ischemia and hemorrhage. Ann Med.

[74] Levy D, Edut S, Baraz-Goldstein R, Rubovitch V, Defrin R, Bree D, et al. Responses of dural mast cells in concussive and blast models of mild traumatic brain injury in mice: potential implications for post-traumatic headache. Cephalalgia. 2015. 10.1177/0333102415617412 PMC5500910 26566937

[75] Herson PS, Traystman RJ (2014). Animal models of stroke: translational potential at present and in 2050. Future Neurol.

[76] Anglemyer A, Horvath HT, Bero L. Healthcare outcomes assessed with observational study designs compared with those assessed in randomized trials. Cochrane Database Syst Rev. Issue 4. Art. No.: MR000034. doi:10.1002/14651858.MR000034.pub2. 10.1002/14651858.MR000034.pub2 PMC8191367 24782322

[77] Fornasari P, Montanari S. Utilizzo del protocollo di minima per l’ictus cerebrale (PMIC) per la valutazione dell’outcome e del percorso riabilitativo di un gruppo di pazienti ricoverati presso l’U.O. di medicina riabilitativa di Cesenatico. Eur Med Phys. 2008;44 (Suppl.1 to No.3).

